# Forced evictions and their social and health impacts in Southern Somalia: a qualitative study in Mogadishu Internally Displaced Persons (IDP) camps

**DOI:** 10.1080/16549716.2021.1969117

**Published:** 2021-09-06

**Authors:** Mohamed Jelle, Joanna Morrison, Hani Mohamed, Raha Ali, Andrea Solomon, Andrew J Seal

**Affiliations:** a Institute for Global Health, University College London, London, UK; b Concern Worldwide Somalia, Mogadishu, Somalia

**Keywords:** Eviction, displacement, humanitarian crisis, IDP, Somalia, land, rents, health and development

## Abstract

**Background:**

Forced evictions are common in conflict-affected settings. More than 500 internally displaced persons (IDPs) are evicted daily in Mogadishu. Context specific research is necessary to inform responsive humanitarian interventions and to monitor the effectiveness of these interventions on IDPs health.

**Objective:**

This study explored the causes of forced evictions and their health impacts among IDPs in southern Somalia.

**Methods:**

We used a qualitative approach, conducting 20 semi-structured interviews, six key informant interviews and four focus group discussions. We used maximum variation sampling to include a wide range of participants and used the framework approach and Nvivo software to analyse the data.

**Results:**

In this context, landlords often rented land without proper tenure agreements, resulting in risk of forced evictions. Informal tenure agreements led to fluctuations in rent, and IDPs were evicted because tenancy laws were inadequate and failed to protect IDP rights. IDP settlements often increased the value of land by clearing scrub, and landlords often sought to profit from this by evicting IDPs at short notice if a buyer was found for the land. The effect of eviction on an already marginalised population was wide ranging, increasing their exposure to violence, loss of assets, sexual assault, disruption of livelihoods, loss of social networks and family separation. Evicted IDPs reported health issues such as diarrhoea, malaria, pneumonia, measles and skin infections, as well as stress, anxiety, psychological distress and trauma.

**Conclusion:**

Forced evictions remain one of the biggest challenges for IDPs as they exacerbate existing vulnerabilities. Prioritizing implementation of legal protection for IDP tenure rights is necessary to prevent unlawful evictions of IDPs. Humanitarian agencies should aim to respond more effectively to protect evictees and provide support to prevent poor health outcomes. Further quantitative research is needed to further examine the relationship between forced evictions and health outcomes.

## Background

Globally, millions of people are effected by forced evictions each yea [1]. Detailed statistics from the Centre on Housing Rights and Evictions (COHRE) reported that over 4 million people were affected by forced evictions between 2007 and 2008, and violence was used in almost 50% of evictions [2]. An absence of formal tenure rights, property market forces, absence of state support for the poor, political conflict, ethnic cleansing and war were the key drivers of forced eviction. Forced eviction often affects the urban poor [3–5], who may be internally displaced. Land rights and legislation may offer little protection to evictees because of patrimonial governance and sometimes legislation is outdated and refers to colonial planning laws and policies that are no longer suitable [6]. The Internal Displacement Monitoring Centre, which was established in 1998 as part of the Norwegian Refugee Council (NRC), is a leading source of data and analysis on internal displacement. It estimates that globally, by the end of 2019, 45.7 million people were displaced within their own countries by armed conflict, violence and human rights violations [7]. Displacement combined with the threat of eviction and forced eviction has severe health effects on affected populations.

Most peer-reviewed research on the health effects of eviction has been conducted in high income countries [8–10]. Two systematic reviews of research on the health effects of eviction included only one study from a low-income country [10,11]. Research in low-income countries has shown that women’s health is particularly affected by forced eviction as they may experience psychosocial distress, miscarriages, gender-based violence, undernutrition, overweight, physical injuries or death [12–15]. Eviction can also contribute to malnutrition and infections such as diarrhoea, respiratory infections, and skin infections in children [3,15]. Qualitative studies suggest that eviction is often experienced by individuals as a personal failure and carries stigma. This can lead to feelings of insecurity, embarrassment, isolation, having a lack of control over key aspects of daily living and the loss of the sense of belonging which can lead to anxiety, depression or suicidal feelings [16–20]. The stress of eviction can also trigger the adoption of unhealthy behaviours particularly during pregnancy such as forgoing meals and prenatal care or engaging in physically demanding work [21].

Somalia has the 6^th^ highest number of IDPs globally [22]. The World Bank estimates the total population of Somalia to be 16 million [23]. Current reports suggest that about 3 million people (19% of the population) live in IDP camps . This is in contrast to other countries in the region like Ethiopia (1.8%), Sudan (5%), Yemen (12%) and South Sudan (12.7%) [22,23]. A high number of IDPs in Somalia live in the outskirts of the capital, Mogadishu, in an area known as the Afgooye Corridor and settle on private land. IDPs in Mogadishu have often fled to escape conflict and natural disasters in rural areas, such as recurrent droughts and floods. Most IDPs in Somalia are from marginalized or minority clans [24,25], which makes them more vulnerable to land grabs, forced evictions, deplorable housing conditions, compulsory relocations, and social and political exclusion than the majority clans [26,27]. None of the IDP camps have formal status, and there are no government or UN-operated IDP ‘camps’ that can provide IDPs with assured shelter, security and services [28]. Data from the Norwegian Refugee Council’s Eviction Information Portal (a dashboard that tracks eviction trends in Somalia) show that in 2019 more than 550 individuals were evicted in Mogadishu every day and that evictions have been increasing over the past few years [29]. At the time of our study, land legislation offered little protection to IDPs as it was outdated and referred to constitutional frameworks that were no longer in use [30,31]. IDPs were mostly evicted by private citizens, informal settlement managers (also known as Gatekeepers) and government officials [29].

Reports from the Somalia Protection Cluster (a co-ordination body with representatives from UN agencies and non-governmental organsiations) show that forced evictions are usually sudden, often carried out at night, and take place without consultation or notice. Usually no relocation land is provided, property is destroyed – sometimes by bulldozers – and no compensation is given. Forced eviction often uses harassment, threats or violence to make people sign agreements [30]. Reporting on twin evictions in 2017, the Norwegian Refugee Council (NRC) noted significant destruction of livelihoods and property, including humanitarian infrastructure such as feeding centres, water collection points, schools, and latrines. Children were separated from their families, and social networks were affected, limiting IDP access to social and economic support from relatives, neighbours and friends. Women reported sexual violence during the eviction and as a result of the eviction [32].

Despite the evidence that forced eviction can exacerbate health vulnerabilities, previous research on the impact of forced evictions in Somalia has focused on the socio-economic impact and protection challenges faced by evictees [29,30,32,33], and few studies examined the possible linkages between eviction and poor health, and none among Somali IDP populations. Therefore, we conducted research to explore the causes of forced evictions and their health impact among IDPs in southern Somalia. We then developed a conceptual framework to make explicit the links between forced evictions and poor health outcomes.

## Methods

### Study setting

The study was conducted in IDP camps located in the Afgooye Corridor in Deyniile district. Deyniile is one of the three districts of Banadir region that hosts most of the IDPs in Mogadishu [24,34]. The camps are informal settlements located on private lands. They are managed by informal settlement managers, also known as gatekeepers. Gatekeepers provide land, security, conflict management, and social services such as assistance with burial and birth arrangements in exchange for payment in cash or in-kind [28].

The camps are often overcrowded, and many lack basic water, sanitation and health services. Most households are female headed and have about six members. Early marriage is common and age at first birth is 17 years. Only 10% of primary carers of children aged below 5 years are literate [34]. During a large influx in an emergency, new arrivals settle in new camps, and register for humanitarian assistance. Thereafter, they often move to new camps which are less crowded, often have better facilities and they can take the opportunity to register for additional humanitarian assistance [35]. Most IDPs in these camps are from marginalised or minority clans from Bay, Bakool, or the Shabelle regions [24,25]. Only about 10% of household heads are in paid work. The average monthly household income is $68 [34].

### Sampling

We used an established nutrition and mortality monitoring system (NMS) [24,34], to identify study participants from seven camps that had experienced forced eviction. We captured the experience of a variety of IDPs through purposive maximum variation sampling [36]. We recruited participants of different genders, age ranges, regions of origin, clans, and camps of residence (Table 1). A Community Health Worker helped to locate IDPs and we sampled to saturation. IDPs identified the camp leaders/gate keepeers who were present at evictions to participate in the study. We recruited six UN and humanitarian agency workers (cluster focal points) to participate in a focus group discussion and one government official in a key informant interview. No one refused to participate.

**Table 1.** Characteristics of the study participants

Participant characteristics	N(%)
*Population group (N = 52)*	
IDP Mothers/carers of children < 5 years Adolescents 15–18 years Men >16 years Older people aged 55 years and over Camp leaders Government/humanitarian agency staff	18(35) 4(8) 13(25) 4(8) 6(12) 7(14)
*Age (years)(N = 52)*	
<25 25–45 >45	7(14) 32(62) 13(25)
*Gender (N = 52)*	
Male Female	27(52) 25(48)
*IDP Camp of residence (N = 45)*	
Bismillahi Ceeldheer Geedoole Gumeysidiid Muxtaad Shaqlane Zabiid	5(11) 3(7) 8(18) 7(16) 7(16) 8(18) 7(18)
*IDP Clan (N = 45)*	
Dir Digil & Mirifle Hawiye Minority	2(4) 17(33) 8(15) 18(38)
*IDP Regions of origin (N = 45)*	
Benadir Bay Lower Shabelle Middle Shabelle Nugal	4(9) 4(9) 34(76) 2(4) 1(2)

### Data collection

**Figure 1.**
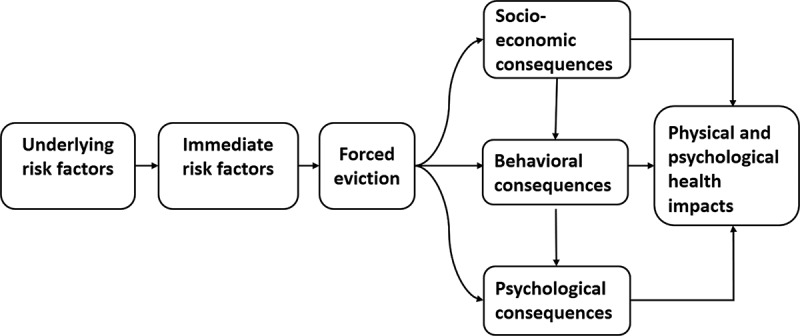
Conceptual framework of potential mechanisms to explain the relationships between forced eviction and poor health outcomes

Data were collected in November and December 2018 by two female Somali researchers (HM and RA) with health backgrounds and prior experience in qualitative data collection. They were trained and supervised by a Somali speaking Kenyan qualitative researcher (MJ). We conducted semi-structured interviews, key informant interviews (KIIs), and focus group discussions (FGD). Those who participated in the FGDs did not participate in interviews. We were informed by the literature on forced migration in the development of our topic guides and a conceptual framework that elucidates the linkages between forced eviction and poor health outcomes [37] (Figure 1). We developed and piloted a topic guide with one woman, adjusting thereafter to increase the focus on the health effects of forced evictions. All participants gave informed verbal consent as literacy levels were low in this population.

We conducted 20 semi-structured interviews (SSI) with 10 women and 10 men. These included 6 mothers/primary carers with children below five years, 6 fathers, 2 male and 2 female adolescents, and 2 men and 2 women who were >55 years of age. SSIs explored the causes of eviction, IDP lived experiences of forced evictions, and how this experience had affected their health and wellbeing. We conducted six key informant interview (KIIs) with camp leaders/gate keepers (three women and three men) of the evicted camps to triangulate information from IDPs. We conducted two FGDs with mothers/primary carers with children below the age of five years old, one with fathers who had been evicted, and one with UN and NGO staff working with IDPs. We discussed the main themes identified from interviews with FGD participants using methodological triangulation to increase the rigor of our study. FGD participants compared narratives of eviction, which made common and diverse experiences explicit.

### Data analysis

Data were digitally recorded and transcribed verbatim into English by HM and RA. Data were reviewed by MJ concurrently with data collection to maintain rigor, encourage interative questioning, and review the data for saturation. MJ also randomly selected and listened to recordings of 10 SSIs and two FGDs to check the quality of translation and transcription. Data were analysed thematically using the framework approach [38]. The research team (HM, RA, and MJ) individually wrote reflexive notes on their initial observations of the data related to the themes and research questions. After data collection was complete, the team re-read the transcripts, discussed their observations on the findings and made an initial coding framework. Each team member coded one or two transcripts with these codes and met to discuss their fit and any adjustments that were necessary. The team then agreed on a set of codes which MJ would apply to all transcripts using NVivo version 11 [39]. Data for each participant type were then charted against themes and methodological triangulation and triangulation between participants was explored. MJ then wrote separate narratives of the experience of men and women and developed recommendations from these narratives.

## Results

**Figure 2.**
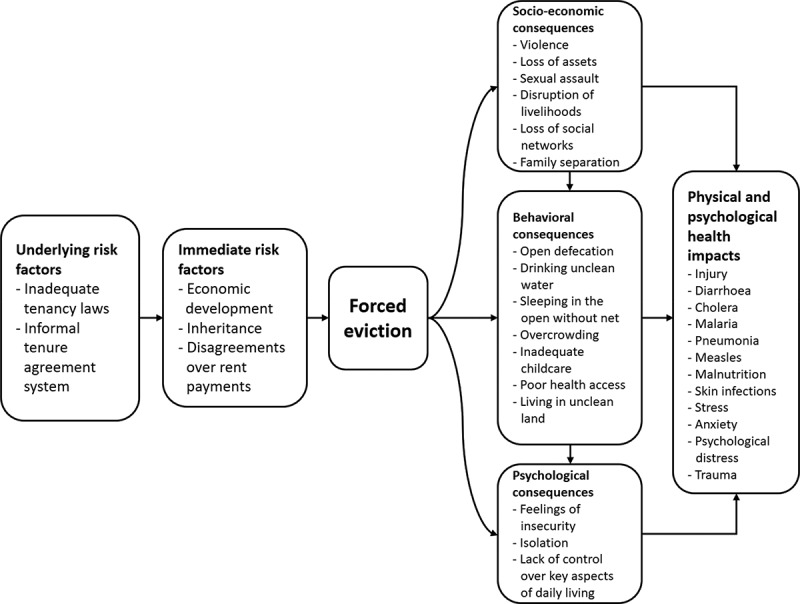
Factors affecting forced eviction and outcomes identified in the qualitative study

We present our findings on the factors affecting forced eviction and their social and health impacts using our conceptual framework (Figure 2). The health problems listed in the framework are people’s experiences of ill health, not diagnoses by health professionals.

### Causes of forced evictions

The lack of tenancy laws and formal tenure agreement system made IDPs feel threatened with forced eviction, and also made them vulnerable to recurrent forced evictions.

When we first settled in this place and rented some land, there was no documentation, everything was verbal and the agreement was made by someone who was representing the landlord. Then after a while the owner himself came and gave us 3 days notice to move out and it was really challenging.

(Interview, a female camp leader).

Most IDPs had been forcibly evicted multiple times. A few IDPs reported that members of minority clans were more vulnerable, but most felt that clan status did not offer protection from eviction.

The lack of formal agreements allowed some flexibility in payment of rent which was linked to the amount of humanitarian assistance that IDPs were receiving, but it did not protect IDPs who were suddenly unable to pay rent. If they were unable to pay their rent in full for a period of time, they risked being evicted.

This land does not belong to us. We have rented it. If we do not pay rent to the landowner or someone else buys it, we will be evicted.

(Interview, an elderly IDP man).

IDPs were also evicted because of increased demand for land for economic development. After settling on a new land, IDPs often clear the bushes and scrub and build shelters, which increases the value of the land. IDPs felt that landowners often allowed them to settle on their land for this purpose, taking advantage of the lack of formal tenure laws and agreements. Sometimes, rather than selling the cleared land, landowners had built new residential or commercial buildings or communal buildings such as mosques. IDPs were also evicted when landowners died and their children or close-family members wanted to divide their inheritance.

It was inherited land and the owners decided that every one of them should take his or her piece of land. We had to move out.

(FGD, an IDP man).

### Loss of assets and livelihoods

IDPs often lose property during evictions. This includes the materials they used to construct their homes as well as household contents such as cooking utensils. Some IDPs earned a living operating small-businesses, such as shops and kiosks, and these structures and their stock were often destroyed in forced evictions. Many IDPs had been living in protracted displacement for several years and evictions destroyed incremental recovery efforts to rebuild lives after displacement. Many families were unable to afford new building materials. Evictions often destroyed infrastructure investments made by humanitarian organisations, such as latrines, water pipes, health centres and schools, which had a significant impact on children’s education.

Household and building materials were lost or broken. We had to buy new materials to build a shelter. There were no toilets and water (in our new place). We had removed and brought the latrines which had been built for us but they were damaged.

(Interview, an IDP mother).

The primary livelihood source within the camps was casual labour, petty trading, and humanitarian assistance from local and international humanitarian organisations. Women often sought daily work such as washing clothes for better-off families and men looked for casual labour in construction. However, during evictions, these established livelihood arrangements were disrupted as families had to spend time dealing with the relocation process, clearing plots and building shelters.

People are exhausted and they lose their possessions. Children go missing. Moreover, we are unable to go for work and earn our living. For instance, mothers can’t go to wash clothes as they are busy moving to the new place and building shelter for the family.

(Interview, an IDP man).

### Loss of social networks

For affected and displaced communities, social networks offer social support and social protection. These networks were disrupted during evictions as social groups were dispersed. Many IDPs found it difficult to adapt to the new locations without the support of their social network.

However, after eviction, some IDPs used their social network to access donations or loans to buy building materials. Women reported that they got support from camp leaders with transportation and financial and in-kind help from people that they worked for. In addition, women said that they got support from other IDP members in the form of donations, loans, food or support with building their shelters. Others said that they would send their children to work after a forced eviction instead of sending them to school.

We didn’t get any assistance except from the camp leader who provided transport to those who could not afford to arrange it. Also the IDP community would help those who could not build their shelters (makeshifts).

(Interview, IDP woman)

We had to adapt to face these challenges. We didn’t get humanitarian assistance but the camp leader helped us with building our shelter (makeshift). We tried our best to cope. For example my little boys would go and do casual work as shoe polishers, my husband is very old and I had to look after the property.

(Interview, IDP woman)

### The impacts of forced eviction on health

Many IDPs live in perpetual fear of eviction whilst struggling to recover from the impacts of displacement, Many IDPs have been displaced multiple times and for periods of over five years. Forced evictions are often violent. IDPs were rarely given sufficient notice to enable them to relocate with dignity, and they were often unable to prevent the loss or destruction of assets. They usually had no say in where they could move to.

Yes, we experienced evictions four months ago. The landowner ordered us to move out and gave us three days’ notice. The eviction was violent and there was fighting that resulted in a mother dying, and another man being injured.

(Interview, an adolescent IDP boy).

Some women had experienced sexual violence around the time of the eviction when shelters were removed and protective social networks were absent. Most of these incidents occurred at night during relocation and mainly affected families who were unable to arrange transport or alternative relocation areas before they were evicted. In the first few days after eviction before they built their makeshifts, women felt vulnerable to rape and sexual violence because they had no shelter. Women had not reported the attacks to the police or to their relatives and neighbours for fear of being stigmatized.

The day that people were moving, there were not enough vehicles to carry us all at once and when the first group of people were dropped off, the vehicle broke and could not come back to carry the rest of us. So I was left behind. My husband works in the city did not come back at that time, so I was all alone when four men came and raped me.

(Interview, an IDP woman).

I was worried and afraid of rape because the place we had settled was remote and bushy. I heard news about a girl that was raped and murdered in another camp so we stayed indoors after sunset.

(Interview, an IDP woman).

In contrast, none of the IDP male participants discussed sexual violence against women. When asked directly if women experienced sexual violence during eviction, they either denied this or referred to women as being more vulnerable to theft when they were evicted:

Yes it (sexual violence) can occur sometimes. For example, if a woman wakes up to go to the toilet after midnight and her husband is away, thieves can come. If there are only women and children in the house theives can try to enter the house and take things.

(FGD, an IDP man)

The sanitation of the new location was often poor, and there were often bushes and scrub that would attract mosquitoes and increase malaria risks for those evicted. Without immediate shelter, IDPs suffered from the heat of the day and cold temperatures at night, which increased children’s risk of illness.

The children are mostly affected. When we were evicted during the day the weather was very hot and we did not have shelter. During the night it was windy and cold, so the children get affected with malaria and cold related illnesses.

(Interview, an IDP man).

In IDP camps, Water, Sanitation and Hygiene (WASH) facilities were usually provided by humanitarian agencies. However, this was not usually the case immediately after an eviction. Open defecation and drinking unclean water were common among newly evicted IDPs which predisposed them to infectious diseases such as diarrhoea or cholera, which could be fatal.

The hygiene and sanitation was very poor when we came here. The place was full of bushes and trees so we had to cut and clean. It took us almost a week to rebuild toilets so we had to defecate outside. That caused health problems such as diarrhoea.

(Interview, an IDP adolescent girl)

Infectious disease outbreaks were common as IDPs were evicted and forced into crowded and unsanitary conditions.

When the eviction happened, we lost many people due to a cholera outbreak. Among them was my 15-year-old niece and five other children.

(Interview, an elderly IDP woman).

Forced evictions increased the burden on caregivers and parents who were struggling to care for their children.

At the time of eviction, household materials were broken. Sometimes the eviction happens unexpectedly and there is no one there to help and no money to buy food for our children. For instance we did not cook food for two days because we were busy relocating the shelter and my husband was sick.

(FGD, an IDP woman)

Evictees were more food insecure during evictions because of the disruption and loss of livelihoods. Livelihoods from shops, kiosks, small businesses and casual labour were lost, which resulted in increased food insecurity. Access to health care was also affected and in the absence of allopathic medicine, many used traditional medicine.

I was sick and bleeding but couldn’t go to a health facility. When I went, I was diagnosed with a tumour in the uterus. One of the children had diarrhoea and haemorrhoids and was treated traditionally by burning it.

(Interview, an IDP woman).

Forced eviction caused stress, despair, anxiety, and psychological distress among IDPs. Many told us that they were constantly worried about the threat of forced eviction, particularly when they were away from their children. Worry about potential separation from children, loss of assets and social networks, risk of violence and rape, and lack of access to health care and food resulted in high levels of stress.

People here are worried and overthink about how they will afford to build a new shelter, or how they will carry the old one and feed their children, as no one will help them. All these worries make them stressed.

(Interview, an IDP man).

## Discussion

Forced eviction in Somalia has reached unprecedented levels over recent years. This qualitative study examined the relationship between forced evictions and poor socio-economic and health outcomes. The inadequacies in existing land laws and tenure agreements to protect IDPs against forced evictions have health, social and economic impacts on this already marginalized population. Forced evictions affected livelihoods, decreased access to healthcare, destroyed social support networks, and led evictees to experience mental and physical ill-health. Women’s health was particularly affected as forced evictions exposed them to higher levels of sexual and physical violence. IDPs had decreased access to maternal health care, and increased food insecurity.

These findings are consistent with other grey literature about the negative social and economic impacts of forced evictions on IDPs in Somalia [30,32,40–42]. There has, however, been very little research in Somalia and in other humanitarian settings, that explicitly acknowledges the linkages between these social and economic impacts and health. A number of conceptual frameworks have been used to examine the relationship between migration and health [37,43,44], however these frameworks fail to elucidate the linkages between forced evictions and health. The conceptual framework presented in this paper makes explicit the independent and interdependent health, social and economic impacts of forced evictions. Many of the health problems listed in our framework such as malaria, diarrhoea, and malnutrition are endemic in Somali IDPs camps and similar settings, but forced evictions are likely to exacerbate these health issues due to the resultant overcrowded living environments, limited access to healthcare, loss of livelihoods, and disruption to water and hygiene services.

### Short term responses

#### Use of moratoriums to stop evictions

Moratoriums – legally authorized periods of suspending forced evictions – have been effective in preventing forced evictions in this context [45]. For example, there have not been any evictions in the months following a moratorium in Baidoa prepared by the municipal authorities in April 2020. The Federal Government of Somalia, Federal States and Banadir Regional Adminsitration (BRA) can prohibit forced evictions through such emergency legislation. As revealed by this and other studies, one of the main causes of evictions is the failure by IDPs to pay rent. Moratoriums should be followed by mechanisms to compensate landowners, for example rent could be included in humanitarian assistance packages. At present, calculations to determine the monthly cash transfer for IDPs do not include rent expenditure, despite the fact that this is a substantial expenditure. The necessity to spend cash transfers on rent is likely to divert spending from health care thus hampering the effectiveness of health and nutrition programmes.

#### Better surveillance and monitoring

Until appropriate laws are instituted and there are improvements in the implementation of eviction laws, the threat of eviction will continue to affect IDPs. Better surveillance and monitoring mechanisms are necessary to enable an informed humanitarian response during and following evictions. This study found no evidence that the most disadvantaged groups (marginalized and minority clans) were disproportionately affected by eviction, despite this being indicated in previous studies [32,33]. It is possible that clan protection of dominant groups could protect women from sexual violence, and facilitate reporting of sexual violence, but we were unable to explore this in our study. Clan protection is an individual’s ability to protect themselve against violence through the fact that they belong to a clan which, by force, can provide deterrence against any attacker [46].

Effective monitoring would enable an analysis of who is being evicted, and the health, social and economic effects of eviction on these groups. Surveillance will help to inform and monitor progress of targeted interventions to help those most affected. Initiatives such as the Eviction Information Portal developed by Norwegian Refugee Council (NRC) and partners are useful in this regard. Our qualitative study findings can be used to inform the development of tools to measure the impacts of forced eviction and indicate the need to further investigate associations between forced evictions and child health outcomes. The study findings are relevant for a range of similar humanitarian settings where IDPs suffer from forced eviction [3,15,47].

#### Targeted immediate support following an eviction

Despite the reported social, economic and health implications of forced evictions, we found that IDPs received little assistance from humanitarian agencies at the time of eviction or immediately afterwards. IDPs relied on camp leaders, relatives, and dispersed neighbours for help. The humanitarian resources allocated to meet the scale of the problem appear inadequate and it is not clear that the protection of evicted vulnerable people is being prioritized. Evicted pregnant women and children in IDPs need support at the time of eviction to enable access to routine primary care, therefore preventing the adverse health effects of eviction.

Studies from high income countries have shown that eviction and eviction threats were significantly related to higher percentages of very low birth weights (VLBW) and infant mortality (IM) among children born in the USA [21,48]. A study from Bangladesh found strong positive association between low birth weight and malnutrition among children under five years old [49]. Other studies have confirmed a relationship between eviction and all cause mortality even after controlling for demographic, socioeconomic, and health conditions prior to the eviction [50]. The humanitarian sector has an important role to play in monitoring new evictions by having early warning systems to enable a timely reponse, and ensure the needs of women and children are prioritised. Our study shows that IDPs require support with transportation, building materials, WASH, health care, and psychological support. In the interim period, health agencies should consider scaling up mobile facilities, including vaccinations and nutrition treatment, to reach children of evicted families.

### Medium term responses

#### Upgrading of existing camps and implementing the national eviction guidelines

Recurrent evictions erode incremental recovery gains by IDPs. It is paramount that the government and other partners should look for alternative durable housing solutions. In the medium term, this could be done by upgrading current settlements by providing IDPs with formal tenure agreements and social services. Implementation of the 2019 National Evictions Guidelines [51], needs to be prioritised to provide a legal framework that will not only protect IDPs against forced evections but will also enable the upgrading of camps. In the absence of a fully functioning, formal judicial system, alternative dispute resolution mechanisms such as the Xeer (Somali customary law) and Sharia law are applied. It is important to engage and develop the capacities of religious, local elders and judges who use these customary justice resolution mechanisms to make them aware of the vulnerabilities, rights and needs of evicted populations.

#### Support to rebuild livelihoods

Our study showed that evictions destroyed livelihoods – damaging property, stock and social networks – which affected the mental health of evictees and made households vulnerable to food insecurity, malnutrition and ill health. Humanitarian assistance should include income-generating opportunities such as microcredits/small loans or grants that enable the local community to restore their lost business, maintain their assets, pay their debts, and improve their living conditions in the long term.

### Long term responses

#### Resettlement schemes

Informal urban settlements are a challenge for both the municipality and the residents. In the long term, a more effective and long-term settlement approach, which is based on international standards, should be explored. IDPs could be resettled on public lands provided by the Government in order to mitigate the risk of forced evictions. This process would not only respect the human rights standards articulated in international conventions concerning the Right to Adequate Housing [52], but also ensures IDPs’ long term potential to contribute to the urban economy.

### Limitations

It was challenging to maintain privacy during data collection, which might have led to underreporting of lack of access to humanitarian assistance, payment of rents, clan related issues, and sexual violence. Whilst maximum variation sampling allowed us to describe the experience of eviction from many perspectives, we were unable to explore the effects of eviction in any depth with specific groups.

## Conclusions

Our study has described the health and social consequences of forced evictions on IDPs in Mogadishu. While forced evictions in humanitarian contexts have generally been viewed from a rights-based approach that focuses on social protection, the effect of eviction on health also needs to be considered. Forced evictions remain one of the biggest challenges for IDPs as they exacerbate existing vulnerabilities. Prioritizing implementation of legal protection for IDP tenure rights is necessary to prevent unlawful evictions of IDPs, but in the meantime, humanitarian agencies should aim to respond more effectively to protect evictees and provide support to improve their health. Our findings also emphasize the importance of setting up better surveillance systems to monitor evictions and their effects. Further quantitative research is needed to further examine the relationship between forced evictions and health outcomes.

## Data Availability

The datasets generated and analysed during the current study are not publicly available due to them containing information that could compromise research participant privacy/consent but are available from the corresponding author on reasonable request.

## Paper context

Forced evictions are common in humanitarian settings but research on their health impact is scarce. Our study explored the health and socio-economic consequences of forced evictions on Internally Displaced Persons in Mogadishu. Implementation of legal protection for IDP tenure rights should be prioritized. Humanitarian agencies should aim to provide targeted support to prevent poor health outcomes. Further quantitative research to assess evictions and their effects is needed to further examine the relationship between forced evictions and health outcomes.
